# Health insurance enrollment strategies during the Affordable Care Act (ACA): a scoping review on what worked and for whom

**DOI:** 10.1186/s13690-021-00645-w

**Published:** 2021-07-12

**Authors:** Angelo Ercia, Nga Le, Runguo Wu

**Affiliations:** 1 Centre for Health Informatics, Division of Informatics, Imaging and Data Sciences, University of Manchester, Manchester, UK; 2 Department of Health & Human Services, County of Marin, Marin, California USA; 3 Institute of Population Health Sciences, Queen Mary University of London, London, UK

**Keywords:** The affordable care act, Medicaid, Private insurance, Insurance enrollment

## Abstract

**Background:**

The Affordable Care Act (ACA) provided an opportunity for millions of people in the U.S. to get coverage from the publicly funded Medicaid program or private insurance from the newly established marketplace. However, enrolling millions of people for health insurance was an enormous task. The aim of this review was to examine the strategies used to enroll people for health insurance and their effectiveness after implementing the ACA’s coverage expansion.

**Methods:**

The PRISMA Extension for Scoping Review (PRISMA-ScR) guided this review. Included studies were empirical studies that met the inclusion criteria and published between 2010 and 2020. Studies were searched mainly from two scholarly databases, CINAHL Plus and Medline (PubMed) using keyword searches. Hand searches from the references of selected journals were also performed. Content analysis was conducted by two authors in which codes were inductively developed to identify themes.

**Results:**

There were 2213 potential studies identified from the search, but 10 met the inclusion criteria. The research design of the studies varied. Two studies were randomized trials, one quasi-experimental trial, three mixed-methods, two qualitative and two quantitative. All studies focused on strategies used to inform and help people enroll for either Medicaid or private insurance from the marketplace. This review identified three key strategies used to help enroll people for coverage: 1) individual assistance; 2) community outreach; and 3) health education and promotion (HE&P).

**Conclusion:**

Community-based organizations were likely to use a combination of the three strategies simultaneously to reach uninsured individuals and directly help them enroll for health insurance. Other organizations that aimed to reach a wider segment of the population used single strategies, such as community outreach or HE&P.

**Supplementary Information:**

The online version contains supplementary material available at 10.1186/s13690-021-00645-w.

## Background

Implementing the Affordable Care Act’s (ACA) coverage expansion in 2014 provided the opportunity for millions of uninsured people in the United States (U.S.) to gain coverage. The ACA was able to accomplish this by simultaneously expanding the publicly funded insurance program, Medicaid, and private insurance through the establishment of the marketplace. The first 5 years of the ACA’s implementation enabled about 10.8 million people to gain coverage from Medicaid and 11.7 million to gain coverage from private insurance [1].

Despite the millions of people gaining insurance from the ACA, the Congressional Budget Office estimated 23 to 26 million people would remain uninsured [2]. Several factors contributed to the ACA’s inability to eliminate uninsurance. One factor was the Supreme Court’s ruling that made Medicaid expansion an option for state governments. Thirty-nine states including the District of Columbia (D.C.) have expanded Medicaid while 12 states have not expanded [3], thus putting many low-income nonelderly adults at risk of remaining uninsured [4]. Purchasing private insurance from the newly established marketplace was an option for nonelderly adults unable to get Medicaid, but some found it unaffordable even with the possibility of receiving federal subsidies [5].

Another factor that may have also contributed to people remaining uninsured was navigating the enrollment process for coverage [6, 7]. Many people had limited understanding of health insurance, the enrollment process [8–10] and misunderstanding of the ACA [11]. The federal government, in collaboration with state and local governments, enacted multiple strategies to help people enroll for coverage. For example, a “no wrong door” enrollment system enabled people to enroll for Medicaid or private insurance on a centralized website [12]. The federal government also established the Consumer Assistance Program to conduct outreach and help people experiencing any problems with the enrollment process or post enrollment issues such as appealing to any denied claims [13]. Some state managed health insurance marketplaces established an assistance program for people to receive guidance on selecting, understanding, and enrolling to an insurance plan [13]. There are many other strategies used by local organizations. However, there is still limited understanding of the strategies used and their effectiveness in helping people enroll for coverage [14]. Therefore, this scoping review aims to identify and synthesize the strategies used during the ACA to help enroll nonelderly adults for Medicaid or private insurance and their effectiveness.

## Methods

### Search strategies and selection criteria

This review explored the strategies used and their effectiveness during the ACA to enroll uninsured people for coverage. The PRISMA Extension for Scoping Review (PRISMA-ScR) guide [15] directed the search for peer-reviewed literature. We conducted our literature search on two scholarly databases, CINAHL Plus and Medline (PubMed) from March to May 2020. The following search terms were used in both databases: “The ACA” [OR] “The Affordable Care Act” [OR] “The Patient Protection and Affordable Care Act” [AND] “enrollment” [OR] “take up” [AND] “health insurance”. We also conducted hand searches from the references of selected journals.

The following are the inclusion criteria for the studies selected for this review: 1) empirical studies that investigated the enrollment strategies used to enroll people for health insurance under the ACA, 2) described research design and methods, 3) focused on nonelderly adults (ages 18–64), 4) studies occurred between January 2010–December 2020, 5) and studies written in English. The following are the exclusion criteria that excluded studies from this review: 1) studies not specifically investigating enrollment strategies used during the ACA’s coverage expansion, 2) studies that primarily focused on enrolling children or elderly adults for coverage, 3) systematic reviews, editorials/ commentaries, grey literature or reports.

### Analysis

AE and NL independently screened the search results by reviewing the title and abstract of each study and assessed whether they met the inclusion criteria. They compared their selections and discussed any discrepancies to meet consensus whether to include or remove them. This process occurred in several iterations to ensure the articles met the aim of the review. The authors then conducted content analysis on the selected articles. The initial process included reading through the literature passages and identifying the enrollment strategies to inductively develop a coding framework. The coding framework included various elements of the enrollment strategies such as their strength, weaknesses, barriers, and limitations. This enabled the team to identify themes.

## Results

**Fig. 1:**
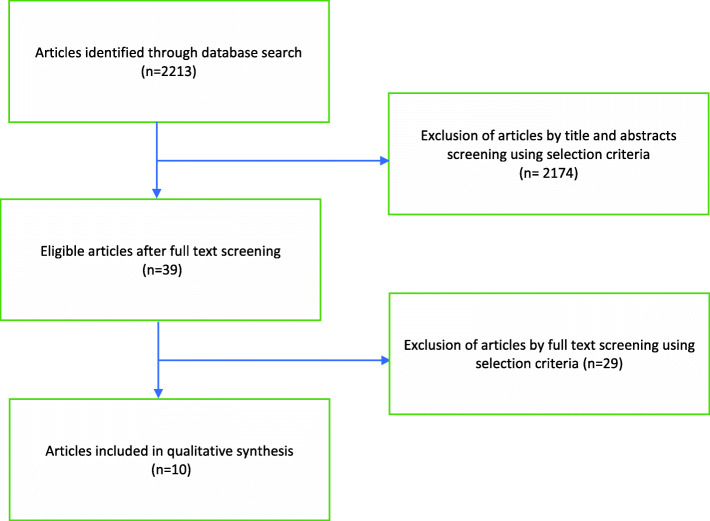
Flow chart of selected articles

The search produced a total of 2213 articles. Ten articles were included in the review as they met the selection criteria (see Fig. 1). The selected articles varied in its research design and included two randomized trials, one quasi-experimental trial, three mixed-methods, two qualitative, and two quantitative studies. Most of the studies investigated strategies used to encourage uninsured individuals to get coverage from the Medicaid program or private insurance from the marketplace. Two studies only investigated strategies to help enroll uninsured individuals for Medicaid. Three studies focused on strategies to enroll people for marketplace private insurance. The supplementary document (Supplementary file 1) summarizes key characteristics of the selected studies, such as study aim, design, target population, insurance coverage, and location.

### Enrollment strategies

We identified three key strategies used to help enroll people for health insurance. These strategies were 1) individual assistance, 2) community outreach, and 3) health education and promotion (HE&P). Specific strategies were not only used to help people enroll for either Medicaid or private insurance. However, some organizations (e.g., Federally Qualified Health Centers and community-based organizations) implemented comprehensive approaches that incorporated aspects of the three strategies. Table 1 provides a summary of the enrollment strategies.

**Table 1:** Description of enrollment strategies from selected studies between January 2010 and December 2020

Author	Location	Description
**Individual assistance**		
Call et al. [16]	Minnesota (statewide)	▪ Located in various locations. ▪ Provided education on coverage options. ▪ Provided support throughout the enrollment process.
Getrich et al. [17]	New Mexico (urban & rural areas)	▪ FQHCs and community health centers, (academic health centers, faith-based clinics) had navigators to provide in person assistance.
McGeehan et al. [18]	New York (New York City)	▪ Social workers determined individuals’ insurance eligibility. ▪ Referred individuals to a facilitated enroller from a local non-profit organization for additional support.
Orzol & Hula [19]	Arizona, Florida, Georgia, Illinois, Michigan, New Jersey, North Carolina, Ohio, Pennsylvania, Tennessee, Texas	▪ Collected individual contact to follow-up and provide information on health insurance coverage, enrollment, and key deadlines. ▪ Collaborated with local organizations to extend reach.
Politi et al. [20]	Missouri (St. Louis region)	▪ Online decision aid called “Show Me My Health Plan” (SMHP). ▪ SMHP aimed to 1) simplify written information and graphics 2) activities to assess understanding of health insurance information 3) Financial calculator for plans 4) assess appropriateness of selected plan based on need.
Raymond- Flesch et al. [21]	California (urban & rural areas)	▪ Provided in-person assistance for people enrolling for coverage (Medicaid or private insurance) and other entitlement programs.
Viramontes et al. [22]	California (southern)	▪ Offered in-person enrollment support.
**Community outreach**		
Call et al. [16]	Minnesota (statewide)	▪ Organized outreach events in community centers, places of worship, townhalls. ▪ Collaborated with small business groups and healthcare providers.
Getrich et al. [17]	New Mexico (urban & rural areas)	▪ Outreach activities that aimed to disseminate information on health insurance. ▪ Outreach events occurred in different forms (e.g., workshops, health sessions) in communal settings.
Orzol & Hula [19]	Arizona, Florida, Georgia, Illinois, Michigan, New Jersey, North Carolina, Ohio, Pennsylvania, Tennessee, Texas	▪ Field outreach to communities in 11 states that did not expand the Medicaid program. ▪ Disseminated information about private insurance.
Viramontes et al. [22]	California (southern)	▪ Organized workshops and community events (e.g., health fairs, 5K races, school site visits) to disseminate information and resources in communities.
**Health Education & Promotion (HE&P)**		
Call et al. [16]	Minnesota (statewide)	▪ Developed educational and promotional materials. ▪ Organized print and social media campaigns.
Getrich et al. [17]	New Mexico (urban & rural areas)	▪ Disseminate information on health insurance in multi-language and various advertisement campaigns.
Marzilli-Ericson et al. [23]	Colorado (statewide)	▪ Enhanced nudging system (included paper-based letters and emails) in the state marketplace website to encourage people to shop and change their plan.
Karaca- Mandic et al. [24]	Nationwide (represented 80% of the US population)	▪ Televised advertisement on health insurance directed to uninsured adults under age 65 during open enrolment. ▪ Advertisement came from various sources: insurance advertisements, political advertisements, and local news coverage.
Orzol & Hula [19]	Arizona, Florida, Georgia, Illinois, Michigan, New Jersey, North Carolina, Ohio, Pennsylvania, Tennessee, Texas	▪ Online media campaigns to inform people about the ACA and resources available on their website to enroll for coverage.
Viramontes et al. [22]	California (southern)	▪ Multi-channel advertisement campaign (e.g., billboards, digital marketing, and radio) to disseminate information about the ACA and enrolling for coverage.
Wright et al. [25]	Oregon (statewide)	▪ Enhanced materials and nudges that aimed to improve Medicaid enrollment. ▪ Developed simplified information on health insurance, enrollment, and deadlines. ▪ Provided generic and personalized messaging (letter, emails, telephone calls) during key deadlines.

#### Individual assistance

Most of the studies identified [16–22] individual assistance as a key strategy used by healthcare providers and community-based organizations to help people enroll for health insurance. The terminology used to describe individual assistance varied. It included terms such as certified application counselors, navigators, community health workers, assisters, enrollment workers, and promotores. While organizations used different terminology, they had similar roles and responsibilities. Assisters aimed to provide in-person education to individuals about their coverage options; screen for insurance eligibility, either for Medicaid or marketplace private insurance; provide assistance and support to complete the application process. Assisters also provided help that went beyond enrolling for coverage such as submitting the correct supporting documents, renewing coverage, and helping individuals change their primary care provider. Some assisters working for Federally Qualified Health Centers (FQHCs) and community-based clinics (non-FQHC clinics) also engaged in community outreach. These outreach events included health education workshops and other face-to-face interactive events that enabled them to disseminate information about health insurance and enrolling for coverage [17].

Some FQHCs and community-based organizations had assistance programs prior to the implementation of the ACA. These organizations expanded their capacity to help more people once the ACA coverage expansion took effect by hiring more assisters [17–22]. Other organizations such as The Weill Cornell Community Clinic (WCCC) utilized their licensed social worker to screen patients for coverage eligibility and collaborated with another community organization to help their patients complete the enrollment process [18].

While FQHCs and community-based organizations used in-person individual assisters to help people enroll for coverage, automated web-based assisters emerged in some state marketplace website. The “Show Me My Health Plan” (SMHP) was an online decision aid system integrated in Missouri’s state-run marketplace website [20]. It helped individuals purchase private insurance in the marketplace by simplifying information and graphics, developed interactive activities to assess understanding of health insurance, and provided individualized report to ease the selection of a plan that met their financial and healthcare needs.

#### Community outreach

Four studies [16, 17, 19, 22] examined the role of community outreach as a strategy to inform and link people to enrollment resources. FQHCs and community-based organizations conducted outreach by organizing community events (e.g., 5 K runs, health fairs), workshops, and community meetings [22]. These events took place in various community locations that included schools, community centers, places of worship, and town halls [16, 17, 22]. Studies did not specifically describe the activities that took place in these outreach events. However, the primary aim was to widely distribute information about the ACA, health insurance, and enrollment resources while building trust and relationship with the communities they served. It also enabled organizations to connect people needing additional help to individual assisters when necessary. For example, the Enroll America program established in multiple non-expanded Medicaid states used community outreach events to connect with individuals to provide information and support to help them with the enrollment process [19].

#### Health Education & Promotion (HE&P)

Individual assistance and community outreach would not be possible without effective health education and promotion initiatives. Seven studies [16, 17, 19, 22–25] highlighted the importance of developing enhanced educational and promotional materials about the ACA policy, health insurance, and the enrollment process. Four studies [16, 17, 19, 22] primarily highlighted the role of developing health education and promotion messages to carry out communication campaigns through different media outlets (e.g., digital, online, print, radio, social media) to increase awareness of the ACA and the opportunity to enroll for coverage. Karaca-Mandic et al. [24] investigated the impact of TV advertisement from different sources (private insurance companies, state, news, political advertisement) and its impact on enrollment.

Two studies [23, 25] investigated a different type of HE&P strategy used in two states. The nudging system aimed to distribute direct enhanced educational and promotional materials through paper-based and/or online platform to individuals. The Colorado state marketplace used the nudging system to encourage individuals to shop and potentially change their plans as necessary. The state of Oregon used a similar system to encourage eligible individuals to enroll for the Medicaid program.

##### Effectiveness of enrollment strategies

The three strategies used to enroll people for coverage had a different level of effectiveness. Table 2 summarizes the advantages and disadvantages of each strategy.

**Table 2:** Advantages and disadvantages of enrollment strategies from selected studies between January 2010 and December 2020

Enrollment strategy	Advantages	Disadvantages
**Individual assistance**		
In-person assistance	▪ Helped individuals understand their options for coverage and get support to complete enrollment process [16–18, 22]. ▪ Offered support and resources to individuals new to health insurance, immigrants and undocumented migrants living in rural and semi-urban areas [17]. ▪ Established trust and respect from individuals [17]. ▪ Provide structured approach to helping individuals enroll for coverage and maintain records on the number enrollees [17, 18]. ▪ Identified knowledge gap on enrollment and the ACA policy, barriers to enrollment, and technical issues [21].	▪ Time consuming [16–18]. ▪ Unable to provide support to everyone in need due to limited capacity [16–18]. ▪ Unable to enroll individuals when technical and operations problems with enrollment system occurs [16, 17, 21, 22]. ▪ Updated training to assisters can be challenging especially with ongoing changes in the ACA and other enrollment policies [17, 21, 22].
Online decision aid	▪ Improve individual knowledge on health insurance, plan selection[20]. ▪ increase individual confidence in selecting a marketplace plan [20]. ▪ Could reduce the need for in-person assistance to enroll for a marketplace plan [20].	▪ Cannot determine specific aspects of an online decision aid (e.g., educational materials, cost calculator, values component) that helped individuals decide a Marketplace plan [20]. ▪ Only used by people living within a 90-mile radius of St Louis City [20].
**Community outreach**		
Community events (e.g., health fairs, 5 K-runs, school visits)	▪ Encouraged populations to learn about health insurance coverage [16, 17]. ▪ Informed populations about resources to help with enrollment [16, 17]. ▪ Helped increase private insurance enrollment in non-expanded state [19]. ▪ Informed populations that were less likely to be informed about their coverage options under the ACA (e.g., young adults, immigrants) [17, 19, 22].	▪ Time consuming [16, 17]. ▪ Dependent on capacity and ability to organize events [17].
Educational events (e.g., workshops, health sessions)	▪ Provided health insurance information and enrollment resources during health-related workshops [17].	▪ Time-consuming and may require multiple sessions for people to absorb the information [17].
**Health Education & Promotion (HE&P)**		
Educational/ promotional materials	▪ Simplified information on health insurance and coverage eligibility for people had a wide reach [16]. ▪ Informed large segment of the population about the ACA [17].	▪ Some people continue to struggle with the enrollment process [16]. ▪ Segment of the populations were not exposed to the educational materials or campaigns [16, 17].
Social media/ online campaigns	▪ Informed people how to access educational materials and resources to enroll for coverage [16].	
Advertisement campaigns (e.g., print, radio, TV ads)	▪ Reach larger segment of the population with minimal resources [17, 24]. ▪ The public positively responded to health insurance information from TV advertisement. High volume of advertisements during open enrollment was associated with increase enrollment for coverage [24].	▪ Require multiple exposure for people to absorb the information [17]. ▪ The source of the information mattered. People can be negatively affected by certain TV ads. Negative political advertisement on the ACA was associated with declining Medicaid enrollment [24].
Nudging system (paper-based or online)	▪ Encouraged people to visit the marketplace website and review the information [23]. ▪ Low-cost tools (letters, emails) were just as effective than higher intensity tools to encourage individuals to enroll for coverage [25].	▪ The enhanced nudging system did not cause many people to switch plan [23]. ▪ Segment of the population (e.g., elderly) may not have access to technology and/or have limited skills to utilize it [23].

#### Individual assistance

Individual assistance helped uninsured individuals with limited or no knowledge of health insurance and the enrollment process for Medicaid or marketplace private insurance [16, 17]. Uninsured individuals that gained coverage were seven more times likely to receive help from an assister than individuals that remained without coverage [16]. By contrast, AltaMed, the largest community health center in Southern California, found that individual assisters helped over 19,000 people to enroll for coverage during the first year of open enrollment and over 15,000 people during the second year [22]. This strategy was effective because individual assisters helped people select an insurance plan and navigate the enrollment process. Assisters’ ability to build trust with individuals was also found to be key factor to the strategy’s success [16, 17, 22]. A limitation for assisters was the amount of time they could dedicate to individuals. For example, social workers in WCCC spent 25 min per patient to discuss their options for coverage [18] while navigators in FQHCs spent an hour per patient [17]. The ability of assisters to help people enroll for coverage also heavily relied on their knowledge of healthcare plans and skill to navigate the enrollment system. Enrollment training varied among organizations and was impacted by constant policy changes (e.g., changes in the ACA, immigration and tax rules) in states [17, 21, 22]. Technical problems with the enrollment system was also a common problem that delayed the process [17, 21, 22]. The online SMHP program of Missouri’s marketplace website was also effective in helping to increase users’ health insurance literacy and their self-efficacy to decide an appropriate coverage plan for their need [20]. As a result, users were less likely to seek help from in-person assisters. A limitation with SMHP, however, was the inability to identify specific functions that helped people decide an insurance plan in the marketplace and whether it eased the enrolment process.

#### Community outreach

Community outreach took on many forms and was effective in disseminating information and resources to the public. This strategy was particularly effective in engaging with specific segments of the populations that were less informed about the ACA and resources such as first-time enrollees for health insurance, immigrants, and the undocumented populations. The MNsure program in Minnesota found that 76% of uninsured individuals that were exposed to their outreach activities sought more information about health insurance [16]. The Enroll America program found that their outreach activities contributed to the increase in private insurance enrollment in states that implemented a federally facilitated marketplace [19]. Rural FQHCs and semi-urban community health centers also discovered that conducting outreach activities enabled them to connect with immigrant populations that experienced significant challenges enrolling for health insurance [17]. A major limitation of this approach was the time commitment and resources needed for organizations to organize events [16, 17].

#### Health Education & Promotion (HE&P)

Similar to community outreach, health education and promotion strategies aimed to disseminate information about the ACA and the opportunity to enroll for coverage. However, this strategy could reach a larger population with minimal resources through multimedia campaigns. Karaca-Mandic et al. [24] found that the source of the information mattered as state sponsored television advertisement was more effective in encouraging people to enroll for coverage during the open enrollment period compared to advertisements from private insurance companies, news, and political advertisements on the ACA. They found that higher volume of political advertisement on the ACA may have discouraged people to enroll for Medicaid. Advertisement from private sponsors (e.g., private insurance plans) were also not found to encourage people to enroll for insurance.

The nudging system was effective in encouraging people with marketplace insurance to access information on state websites to consider changing their insurance plan [23]. Medicaid eligible individuals benefitted from receiving information and reminders to enroll for the program [25]. An advantage with this strategy is its relatively low-cost and minimal resources to develop. For example, the nudging system that used low-cost strategies such as sending generalized letters, postcards, emails, and automated telephone calls were just as effective as personalized and higher cost strategies that included in-person outreach and individualized assistance in encouraging people to enroll for either coverage type [23, 25]. A disadvantage of an online nudging system is not everyone has access to technological devices to get the information and access the resources. It may also favor certain population as younger people were more likely to respond to online nudges compared to older people, as they may have better access to computer and emails [23].

## Discussion

Implementing the ACA’s coverage expansion provided the opportunity for many uninsured people to gain health insurance. However, enrolling millions of people for coverage was a monumental task. This review aimed to determine the strategies used during the ACA’s coverage expansion and their effectiveness in enrolling people for health insurance. We found the three key strategies used to help enroll people for coverage were: 1) individual assistance, 2) community outreach, and 3) health education and promotion (HE&P).

Individual assisters aimed to provide information and direct support to uninsured individuals that had limited or no knowledge of getting health insurance under the ACA. While they could only reach a finite number of people, their role was essential because they were directly helping people to enroll for coverage. Our findings are supported by Artiga et al. [12] that found individual assisters had an essential role in helping people enroll for Medicaid in four states (Colorado, Connecticut, Kentucky, and Washington) that expanded the program. Artiga et al. [12] and the findings of this review also suggest that assisters are well suited to help people enroll for coverage because they could develop strong ties with the community and act as a broker during the enrollment process. In this role, they could identify problems with the enrollment process and quickly find solutions. While assisters were effective in helping enroll people for coverage, this strategy required extensive resources. Organizations needed to hire more staff to be assisters, provide ongoing enrollment training, and updated information to reflect constant changes in the ACA.

A plausible alternative to in-person individualized assistance is a web-based system. The online decision tool in Missouri’s marketplace website was found to help increase people’s literacy on private insurance and their confidence in choosing a plan. Insurance plan selection could be stressful, as it requires people to select an affordable plan that meets their healthcare service needs within the year [26]. Therefore, the decision tool may be useful to people that need help with selecting a plan but could complete the enrollment process themselves. A study conducted by Cosineau et al. [14] also found the usefulness of a technology-based assister to improve children’s enrollment to California’s Children’s Health Insurance Program (CHIP) as it increased the enrollment by 10 to 11%.

We found the two other strategies to be similar in their aims, but focused on reaching wider segments of the population to disseminate information and resources. Unlike individual assisters, the two strategies primarily aimed to encourage people to get health insurance rather than providing direct enrollment assistance. Community outreach was a strategy used to disseminate information about the ACA and resources on enrollment to the public. The strength of this approach was its focus on reaching specific populations by organizing interactive events that took place in community settings. Other studies have found this approach to be particularly important in reaching underserved populations [12, 27]. Uninsured low-income and underserved individuals may not be engaged with any healthcare providers; therefore, outreach events must occur in locations where they conduct daily activities and socialize [27]. A limitation with this strategy, however, is the scale of its reach. Smaller events such as workshops could reach a handful of people with minimal resources. Larger events might reach hundreds of people with 5 K runs or community events in town halls, but require more resources and collaboration with multiple organizations.

The HE&P strategy may be an alternative to community outreach when the aim is to reach a wider and diverse segment of the populations with minimal resources. Disseminating information is also possible through multiple communication platforms. While disseminating HE&P information was found to be an effective way to encourage people to enroll for coverage, this review also found that the source of the information mattered. Information from certain sources, such as the state government, was more accepted by the public than from the federal government and private insurance. Information with a political agenda was less attractive to the public. It suggests that information from certain sources such as individual assisters or the state government may be more accepted by the public as they have built trust with the community and the public. They may also be seen as a trusted source of information as they aim to provide support to people enrolling for coverage. Artiga et al. [12] also found this in their study as stakeholders from Kentucky and various regions in Colorado, Connecticut and Washington believed state-branded HE&P were important particularly when reaching individuals that were politically unsupportive with the ACA or had limited trust with federal government programs.

### Implication and limitation of the study

The findings of this review suggest that there were three key strategies used to help enroll the public for health insurance under the ACA. Each strategy had its advantages and disadvantages, but combining all of them provided a comprehensive strategy that could reach individuals, specific groups, and the wider population. Because of the complexity of the enrollment process in the U.S. and the limited window period to enroll for health insurance coverage every year, implementing a combination of enrollment strategies might be more effective than a single strategy, especially for regions with diverse population with different needs. The findings of this review suggest that certain strategies are more effective for a specific segment of the population. For example, uninsured people that never had or have limited knowledge of health insurance and the enrollment process may benefit from receiving in-person help from an individual assister. In-person assistants were necessary for some individuals as they needed additional support to understand the benefit, cost, and use of health insurance and navigating the enrollment process. People that are familiar with health insurance and the enrollment process may need less support but benefit from an online assister system that provides simplified information and reminders of key dates to enroll for coverage. Community outreach strategies may be best used to disseminate information to specific segments of the population. By contrast, HE&P strategies could better reach a wider and diverse population with minimal resources. Therefore, organizations that aim to implement strategies to help people enroll for coverage must consider their target population, capacity and resources.

This review aimed to assess the strategies used to enroll people for health insurance under the ACA by assessing peer-reviewed studies. A limitation of this review is the few studies that specifically investigated the strategies used during the ACA to help enroll people for health insurance coverage. More studies looked at the impact of expanding coverage rather than understanding the process of informing people about the opportunities on getting health insurance and enrolling them. A second limitation is the study design of the studies included in this review. Most studies were quasi-experimental case studies. This provided insight on the type of activities organizations implemented and their potential impact to enrollment. However, it was not possible to correlate the implemented strategies to the proportion of people they enrolled for coverage. There needs to be a more rigorous evaluation of specific strategies’ effectiveness. Last, a limitation of this scoping review aimed to summarize the strategies used to enroll people for coverage rather than critically appraised and evaluate. As a result, a risk of bias must be considered with the evidence presented [28]. The implications presented in this review merely offer suggestions for considerations when implementing enrollment strategies.

## Conclusion

This review found that three strategies were primarily used to assist uninsured individuals to enroll for coverage under the ACA: 1) individualized assistance, 2) community outreach, and 3) health education and promotion (HE&P). Selected strategies were not exclusive to helping people enroll for specific coverage type. The findings suggest that the three strategies worked well on informing people about the ACA and the opportunity to gain coverage from Medicaid or private insurance. However, certain strategies may be more effective in providing support with the enrollment process to specific populations.

## Supplementary Information

**Additional file 1:.** Characteristics of selected studies.

## Data Availability

The data used for this study is available from the detailed reference list. No additional data was used for this study.

## Abbreviations

ACA: Affordable Care Act
FQHC: Federally qualified health center
HE&P: Health education and promotion
WCCC: Weill cornell community clinic
SMHP: Show me my health plan
U.S.: United States
