# Supplementary material for: Health insurance enrollment strategies during the Affordable Care Act (ACA): a scoping review on what worked and for whom
Source: Arch Public Health. 2021 Jul 12;79:129. doi: 10.1186/s13690-021-00645-w (PMC8274016; doi:10.1186/s13690-021-00645-w)
Supplement: Supplementary file 1 — Additional file 1:. Characteristics of selected studies. [file 13690_2021_645_MOESM1_ESM.docx]

**Supplementary file 1**

Supplementary file 1: Characteristics of selected studies

| Author (Year) | Study aims | Study design | Target population | Insurance coverage | Location |
| --- | --- | --- | --- | --- | --- |
| Calls et al. [16] | Determined whether and how individuals most likely eligible to gain coverage from the ACA obtained it in the first year of implementation. | Longitudinal; Mixed- methods  Utilized 2013 Minnesota Health Access Survey (MNHA) and interviews | Adults eligible for Medicaid or Marketplace plans | - Medicaid - Private insurance from marketplace | Minnesota (statewide) |
| Getrich et al. [17] | Investigated the enrollment experience of Federally Qualified Health Centers (FQHCs) clinicians and non-clinicians in New Mexico serving high population of immigrant patients. | Qualitative  Conducted interviews and participants observation during 2013 and 2014 | Adults eligible for Medicaid or Marketplace plans | - Medicaid - Private insurance from marketplace | New Mexico (urban and rural areas) |
| Marzili-Ericson et al.[23] | Tested the effects of two types of nudging system from the standard messaging within the state-operated marketplace to determine the likelihood of consumers shopping for a plan. | Randomized controlled trial | Eligible enrollees for marketplace plans | Private insurance from marketplace | Colorado (statewide) |
| McGeehan et al. [18] | Assessed the effectiveness of the enrollment process utilized in a student run free clinic (SRFC) in New York City. | Mixed- methods  Interviews  Chart review | Adults eligible for Medicaid or Marketplace plans | - Medicaid - Private insurance from Marketplace | New York City, New York |
| Karaca- Mandic et al. [24] | Investigated the impact of broadcast health insurance advertisement from county level data and enrollment for coverage. | Quantitative | Adults eligible for Medicaid or Marketplace plans | - Medicaid - Private insurance from Marketplace | Nationwide- represented 80% of the US population |
| Orzol & Hula [19] | Evaluated the impact of Enroll America’s outreach strategy to support enrollment in Marketplace coverage. | Quasi-experimental design  Compared cross-state enrollment to estimate the impact of field outcome where Enroll America was present | Eligible enrollees for marketplace plans | Private insurance from Marketplace | Arizona, Florida, Georgia, Illinois, Michigan, New Jersey, North Carolina, Ohio, Pennsylvania, Tennessee, Texas |
| Politi et al. [20] | Determined the effectiveness of the Show Me My Health Plans (SMHP) program role in improving people’s ability to enroll for marketplace insurance. | Quantitative | Eligible enrollees for marketplace plans | Private insurance from Marketplace | St Louis region, Missouri |
| Raymond- Flesch et al. [21] | Determined effective enrollment strategies of California’s “No Wrong Door” policy” from the perspective of California Medicaid government and community-based enrollment workers. | Qualitative  Interviews  Focus groups | Eligible enrollees for Medicaid | Medicaid | California- statewide (urban and rural areas) |
| Viramontes et al. [22] | Described the effectiveness of the enrollment process practiced in Alta Med, a large community health center. | Mixed methods  Interviews  Secondary data analysis if patient’s enrollment | Adults eligible for Medicaid or Marketplace plans | - Medicaid - Private insurance from Marketplace | Southern California |
| Wright et al. [25] | Evaluated the effectiveness of nudges and enhanced communication to encourage Medicaid enrollment. | Randomized control design | Eligible enrollees for Medicaid | Medicaid | Oregon |
